# Left atrium decompression devices across the spectrum of ejection fraction in heart failure: an updated systematic review and meta-regression analysis

**DOI:** 10.1007/s10741-023-10317-2

**Published:** 2023-05-10

**Authors:** Christian Basile, Stefania Paolillo, Paola Gargiulo, Federica Marzano, Santo Dellegrottaglie, Vincenza Abbate, Antonio Ambrosio, Francesca Carbone, Simona Dell’Aversana, Immacolata Esposito, Maria Francesca Fierro, Pasquale Perrone-Filardi

**Affiliations:** 1 Department of Advanced Biomedical Sciences, Federico II University of Naples, Naples, Italy; 2 Villa dei Fiori Clinic, Corso Italia, 80011 Acerra, Naples, Italy

**Keywords:** Heart failure, Interatrial shunting, Meta-analysis, Interventional heart failure treatment, Health-related quality of life outcomes

## Abstract

**Background:**

In patients affected by heart failure (HF) with reduced ejection fraction (HFrEF), pharmacological treatments have been proven to alleviate symptoms and improve prognosis, while no treatment other than sodium-glucose co-transporter-2 inhibitors have demonstrated significant effects in HF with preserved ejection fraction (HFpEF). Left atrium decompression devices (LADd) have been recently investigated as a new interventional approach in patients with HFpEF.

**Objectives:**

To assess the efficacy of LADd on soft endpoints in HF patients across the spectrum of ejection fraction.

**Methods:**

PubMed and Web of Science were searched without restrictions from inception to 28 May 2022 to identify valuable articles. The studies that met the inclusion criteria were analyzed. The prespecified main outcomes were the change from baseline in 6-min walking distance (6MWD), NYHA class and health-related quality of life (HRQoL). Secondary outcomes were reduction in HF hospitalizations, echocardiographic, and hemodynamic parameters.

**Results:**

Eleven studies, with a total of 547 patients, were included. LADd significantly improved 6MWD by 43.95 m (95% CI 29.64–58.26 m), decreased NYHA class by 0.93 (95% CI 1.20–0.67), and improved HRQoL questionnaire by 20.45 points (95% CI 13.77–27.14) with better results for all outcomes in patients with lower EFs.

**Conclusion:**

The present meta-analysis suggests that LADd are favorable in improving 6MWD, NYHA class, and HRQoL in HF across a wide spectrum of ejection fraction, with better outcomes in patients with lower EFs.

**Trial registration:**

CRD42022336077, URL: https://www.crd.york.ac.uk/prospero/display_record.php?RecordID=336077.

**Supplementary Information:**

The online version contains supplementary material available at 10.1007/s10741-023-10317-2.

## Introduction

In patients affected by heart failure (HF) with reduced ejection fraction (HFrEF; left ventricular EF ≤ 40%), pharmacological treatment has been proven to favorably affect cardiac remodeling, alleviate symptoms, and enhance cardiac function and prognosis [1]. On the contrary, apart from sodium-glucose co-transporter-2 inhibitors (SGLT2i) [2, 3], no pharmaceutical treatment has been demonstrated to reduce morbidity and mortality in individuals with HF with preserved ejection fraction (HFpEF; EF ≥ 50%). A common symptom of patients with HF, regardless of EF, is dyspnea on efforts, which may be the result of an abrupt development of pulmonary congestion, due to elevated left ventricular and atrial filling pressures [4]. Individual titration of HF pharmacological treatment, especially diuretics, guided by invasive pulmonary artery pressures analysis, may better control congestion and minimize HF hospitalizations [5]; however, pressure-guided HF therapy requires patient adherence, and it is rather difficult and expensive [6]. Similarly to patients with mitral stenosis, in whom a concomitant small congenital atrial septal defect (Lutembacher’s syndrome) is associated with fewer symptoms and better outcomes compared with isolated mitral stenosis [7], various left atrium decompression devices (LADd) are currently under investigation in patients with HF. The present systematic review and meta-regression analysis investigated the feasibility and efficacy of dedicated LADd on symptoms, quality of life, functional status, hemodynamic parameters, and cardiac function in HF individuals across the spectrum of EF (Graphical abstract).

## Methods

This meta-analysis was performed based on the Preferred Reporting Items for Systematic Reviews and Meta Analyses guidelines [8] (Table S1) and registered on PROSPERO (CRD42022336077).

### Search strategy and study selection

PubMed and Web of Science were searched without any restrictions from inception to 28 May 2022. The search strategy is included in the Supplementary Materials. Two authors separately examined the titles and abstracts of all obtained publications to exclude clearly unrelated research. According to the inclusion criteria, the remaining articles were chosen for full-text examination. The final list of included studies was then reviewed by the authors, and any differences were addressed via discussion. Studies were included if they satisfied the following criteria: (1) a comprehensive study design with rigorous inclusion and exclusion criteria; (2) participants affected by HF; and (3) if a main study had more than one follow-up, only those having the longest follow-up duration were included.

### Data extraction and quality assessment

The primary efficacy outcomes were improvement in six-minute walking distance (6MWD), NYHA class and health related quality of life (HRQoL), assessed by specific questionnaire.

The secondary efficacy outcomes were HF hospitalization (HHF), analyzed by comparing the number of HHF occurred in the follow-up period after LADd implantation to the number HHF occurred in the same time lapse before the device implantation, mean right atrial pressure (mRAP), mean pulmonary artery pressure (mPAP), tricuspidal annular plane systolic excursion (TAPSE), pulmonary capillary wedge pressure (PCWP), NT-proBNP variations, and safety concerns of LADd. Device-related adverse events were considered as safety primary outcome, a composite of device embolization, migration or removal, stroke (fatal and non-fatal) or transient ischemic attack, cardiac tamponade, and emergency cardiac surgery; this outcome was derived according to the events reported in each study as device-related.

In addition, a second safety outcome was analyzed, by only considering randomized controlled trial (RCT) that reported a composite of major adverse cardiovascular, cerebral, and renal events (MACCRE), specifically including cardiovascular death, embolic stroke and new onset or worsening kidney function.

The following information was gathered from each included study: baseline characteristics of studies (authors, publication year, journal, country), patients’ characteristics (sample size, gender, age, comorbidities), hemodynamic variables (post-capillary wedge pressure, mean atrial pressure, tricuspid annulus plane excursion, mean pulmonary arterial pressure), functional status changes (6MWD, NT-proBNP, HRQoL), and major adverse events (all-cause mortality, HHF). To analyze the risk of bias, the GRADE tool was used [9] (Table S2). Two reviewers independently estimated means and measures of dispersion from figures in the reports using DigitizeIt, version 2.5 (Braunschweig, Germany), if required. The final values were determined by averaging the opinions of independent reviewers.

### Statistical analysis

STATA 17.0 (Stata Corp, College Station, TX, USA) was used. The chi-square test and *I*^2^ test were used to investigate heterogeneity, with *p* ≤ 0.10 or *I*^2^ > 50% indicating considerable heterogeneity. A DerSimonian and Laird random-effects model was used. Risk ratios (RR) and 95% confidence intervals (CI) were estimated for binary variables and weighted mean difference (WMD) and 95% CI were determined for the quantitative variables.

In addition, sensitivity analysis, funnel plots, and Egger’s test were performed to assess the stability of estimates and publication bias of included papers. A two-tailed *p* value of 0.05 was deemed significant.

Random-effects meta-regression analysis was performed to measure the impact of baseline ejection fraction on the effect size for the primary outcomes.

As with prior research using comparable analytic methodologies [10], the pre-procedure patient cohort was designated as the comparison group.

Recent methods [11–13] were used to convert data provided as sample size, median, first and third quartiles, or minimum and maximum to mean and its related standard error.

The HRQoL was evaluated by the Minnesota living with heart failure (MLWHF) questionnaire or by the Kansas City cardiomyopathy questionnaire (KCCQ), as both instruments demonstrate HRQoL improvement and deterioration on oppositely directed axes, the MLWHF scores were inverted (higher scores imply superior HRQoL) prior to standardization.

## Results

### Study characteristics

Of 1200 identified papers, 25 were retrieved for a more detailed evaluation (Fig. S1). According to the inclusion criteria, 2 studies were rejected, while 12 paper reported the results of the same trial at different follow-up duration or were abstracts on the same trial. Eventually, 11 studies were included, published between 2015 and 2022, comprising 547 patients, and with a follow-up time that varied from 3 to 27 months [14–24].

Two trials [14, 16] assessed quality of life by the MLWHF questionnaire and 6 studies [15, 17, 19, 20, 23, 24] by the KCCQ; the two questionnaire scales were converted in order to compare the results, as previously described.

Table 1 provides a summary of the baseline characteristics of the included studies.

**Table 1:** Baseline characteristics of the included studies

Author	Year	Study type	Device	Months of follow-up	Patients	Female (%)	Age (± SD)	CAD (%)	HT (%)	DM (%)	AF (%)	NYHA class III (%)	Mean EF (± SD)
Malek et al	2015	Prospective non-randomized	IASD, DC Devices	12.00	11.00	55.00	70.00 (± 11.90)	36.00	91.00	45.00	36.00	81.80	57.00 (± 9.00)
Del Trigo et al	2016	Prospective non-randomized	V-Wave, first gen	3.00	9.00	10.00	62.00 (± 8.00)	90.00	70.00	70.00	70.00	100.00	24.50 (± 8.30)
REDUCE LAP-HF	2016	Prospective non-randomized	IASD II, Corvia Medical	6.00	64.00	34.37	69.00 (± 8.00)	23.00	81.00	34.00	61.00	73.00	47.00 (± 7.00)
REDUCE LAP-HF I	2018	RCT	IASD II, Corvia Medical	12.00	22.00	36.00	69.60 (± 8.30)	47.60	81.80	54.50	54.50	100.00	59.90 (± 9.00)
Rodes-Cabau	2018	Prospective non-randomized	V-Wave, first gen	27.00	38.00	8.00	66.00 (± 9.00)	79.00	84.00	68.00	53.00	97.00	50.00 (± 9.00)
Guimares	2020	Prospective non-randomized	V,Wave, second gen	12.00	6.00	20.00	68.00 (± 9.00)	80.00	70.00	50.00	60.00	100.00	34.00 (± 12.00)
Simar et al	2020	Prospective non-randomized	LA-to-CS	7.00	8.00	18.00	79.66 (± 4.02)	62.50	75.00	0.00	87.50	87.50	53.15 (± 4.00)
PRELIEVE study	2021	Prospective non-randomized	AFR, Occlutech	12.00	53.00	41.00	68.20 (± 8.80)		72.00	49.00	49.00	93.00	
RAISE trial	2022	Prospective non-randomized	NoYa system	6.00	10.00	60.00	60.10 (± 14.70)	0.00	40.00	30.00	20.00	40.00	62.82 (± 2.40)
REDUCE LAP-HF II	2022	RCT	IASD II, Corvia Medical	12.00	309.00	64.00	72.30 (± 7.44)	26.00	89.00	37.00	50.00	77.00	60.00 (± 7.44)
Shang et al	2022	Prospective non-randomized	D-shant	6.00	6.00	50.00	57.83 (± 12.66)		33.00	66.00	0.00	50.00	27.60 (± 13.13)

*AF* atrial fibrillation, *BMI* body mass index, *CAD* coronary artery disease, *DM* diabetes mellitus, *EF* ejection fraction, *HT* hypertension, *NYHA* New-York heart association, *RCT* randomized controlled trial

### Primary outcomes

**Fig. 1:**
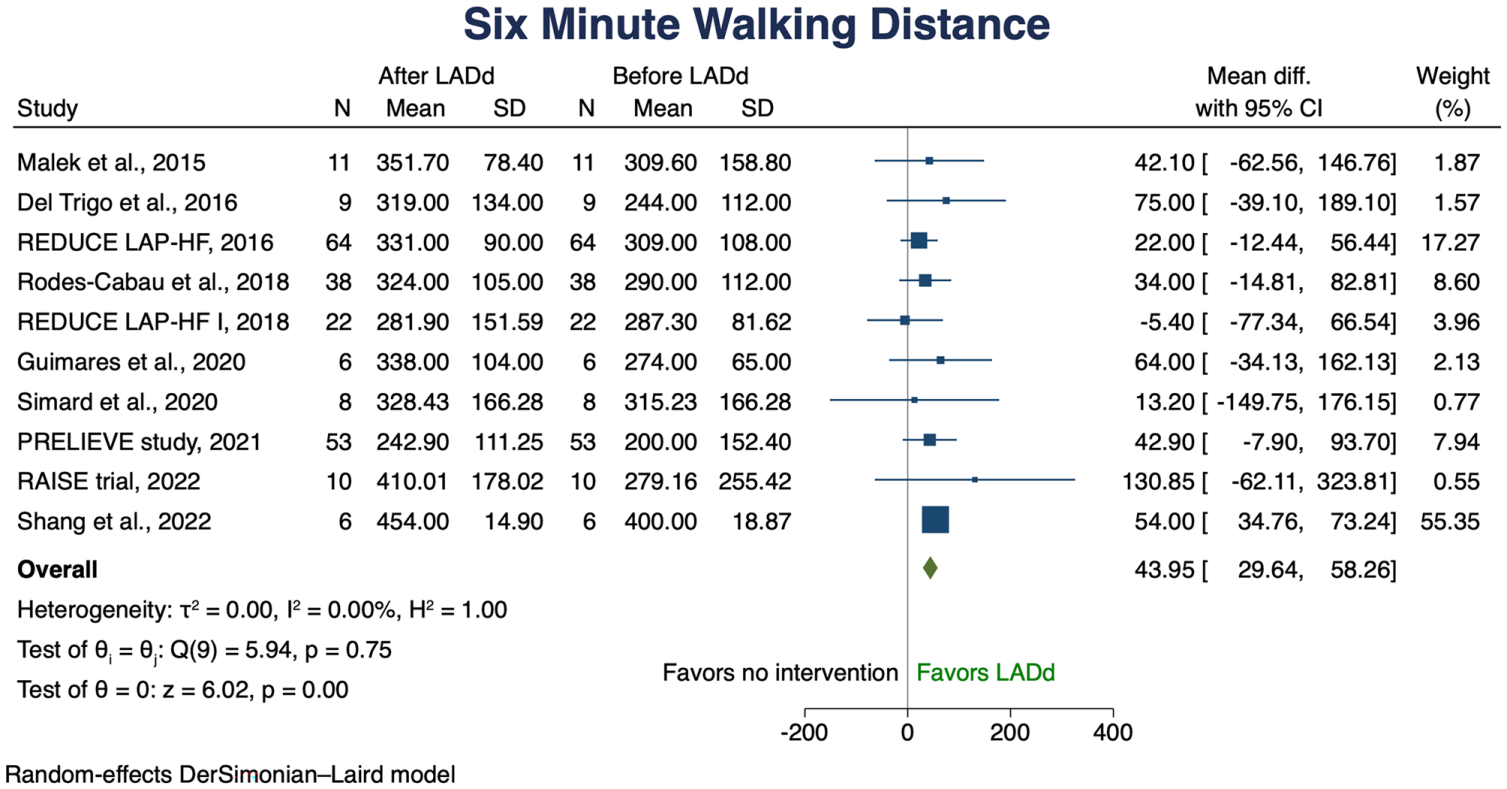
Change in 6MWD (mt). Solid squares represent mean differences in trials and have a size proportional to the weight of the difference. The 95% confidence intervals (CI) for individual trials are denoted by lines and those for the pooled mean differences by empty diamonds. 6MWD, six-minute walking distance; CI, confidence interval

**Fig. 2:**
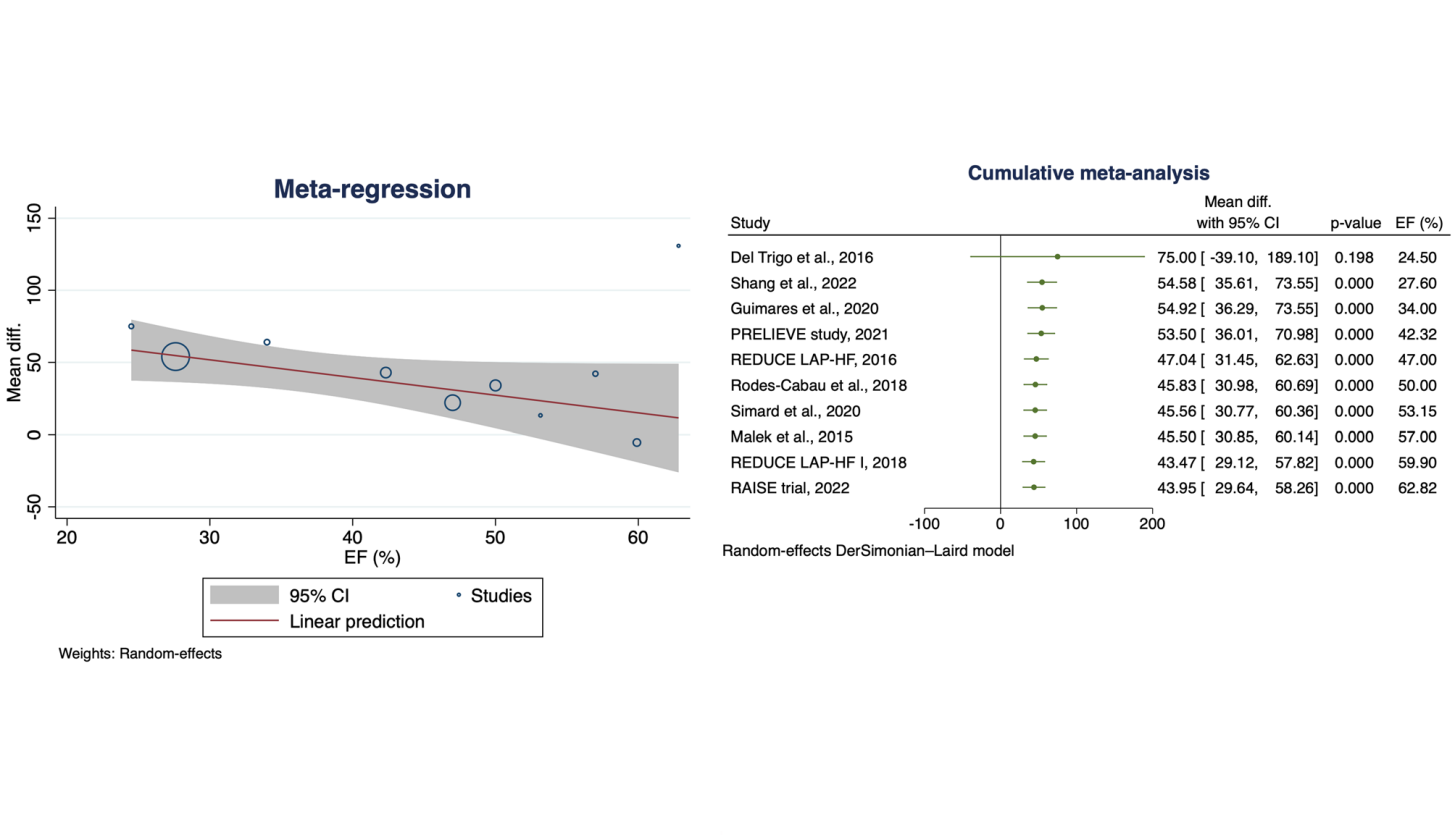
Meta regression analysis (on the left) and cumulative meta-analysis (on the right) for 6MWD change with EF as moderator. 6MWD, six-minute walking distance; CI, confidence interval

Ten studies examined the change in submaximal exercise capacity assessed by the 6MWT. The mean baseline 6MWD varied between 242 and 454 m. Among the included studies, the use of LADd provided an average mean 6MWD increase of 43.95 m (95% CI 29.64–58.26 m) (Fig. 1). To further investigate this outcome result, meta regression and cumulative meta-analysis were performed according to EF (Fig. 2). Meta regression analysis showed a consistent effect across the spectrum of EF, with a greater effect in low EFs; cumulative meta-analysis confirmed this observed trend.

**Fig. 3:**
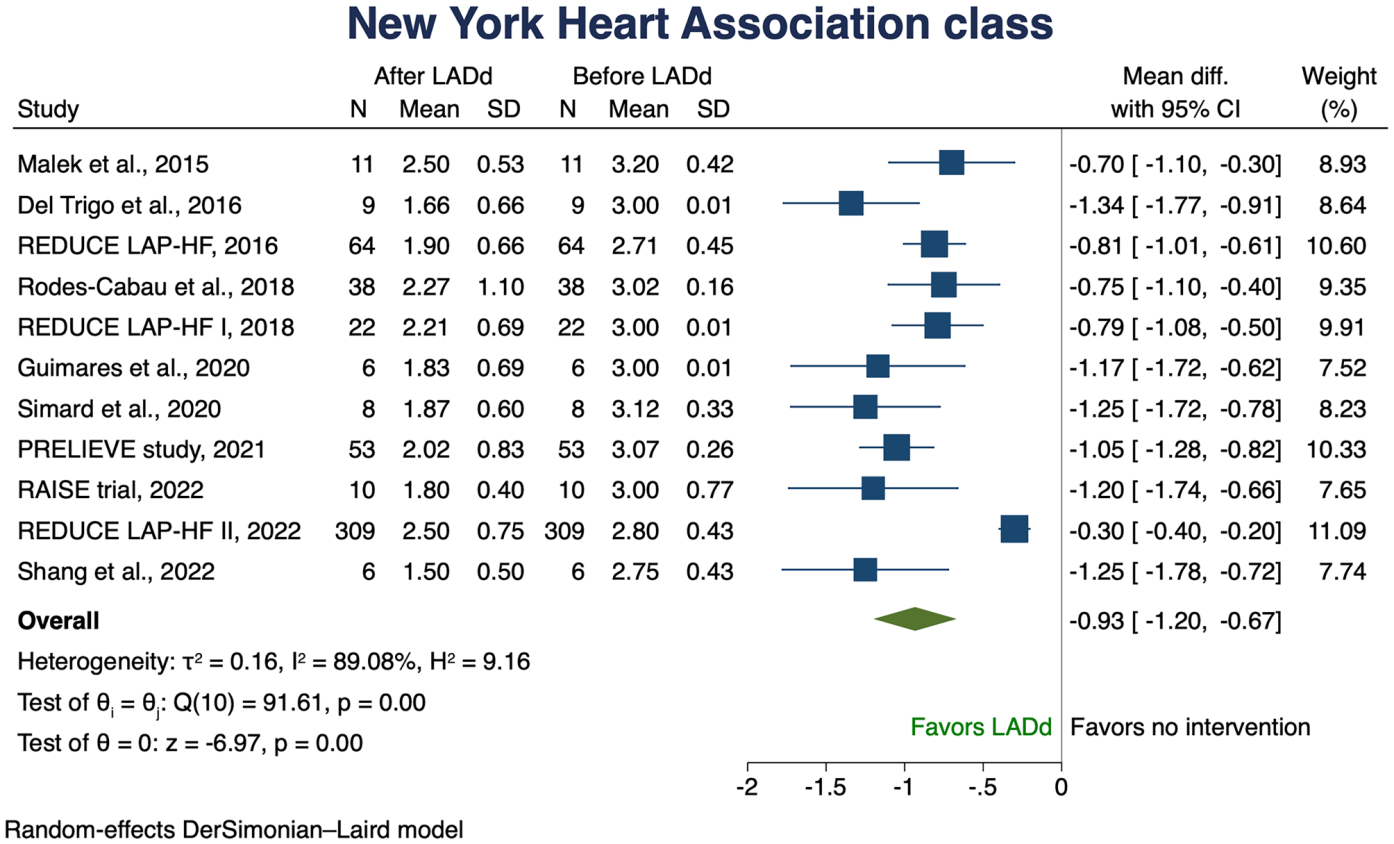
Change in NYHA class. Solid squares represent mean differences in trials and have a size proportional to the weight of the difference. The 95% confidence intervals (CI) for individual trials are denoted by lines and those for the pooled mean differences by empty diamonds. CI, confidence interval

**Fig. 4:**
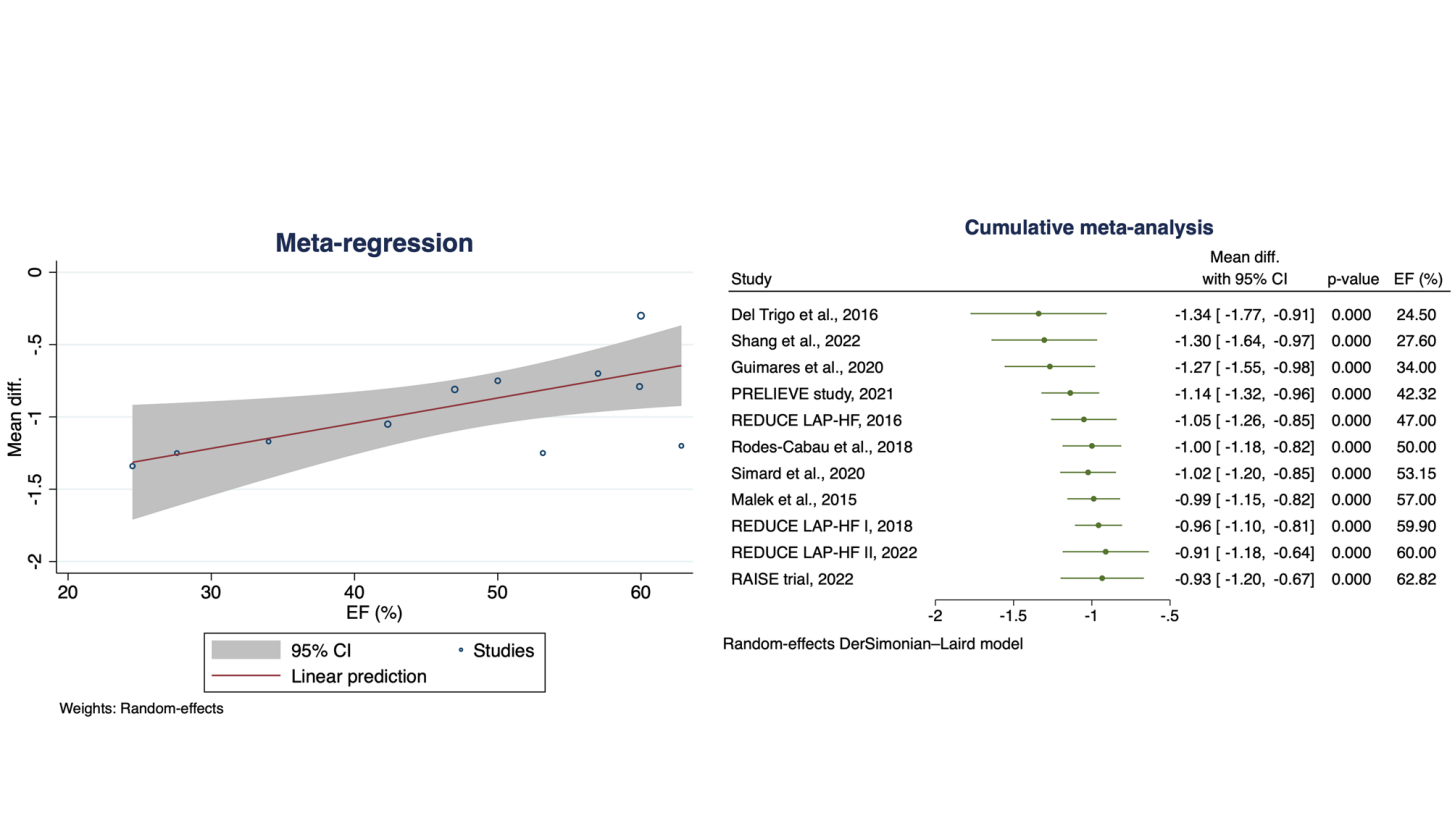
Meta regression analysis (on the left) and cumulative meta-analysis (on the right) for NYHA class change with EF as moderator**.** CI, confidence interval

Eleven studies examined the change in NYHA class with the use of LADd; the average mean NYHA class decreased by 0.93 (95% CI 1.20–0.67) (Fig. 3). To further explore this finding and to assess the considerable reported heterogeneity, meta regression and cumulative meta-analysis were performed according to EF (Fig. 4). Meta regression analysis showed a consistent effect across the spectrum of EF, with a greater effect in low EFs; cumulative meta-analysis confirmed this observed trend.

**Fig. 5:**
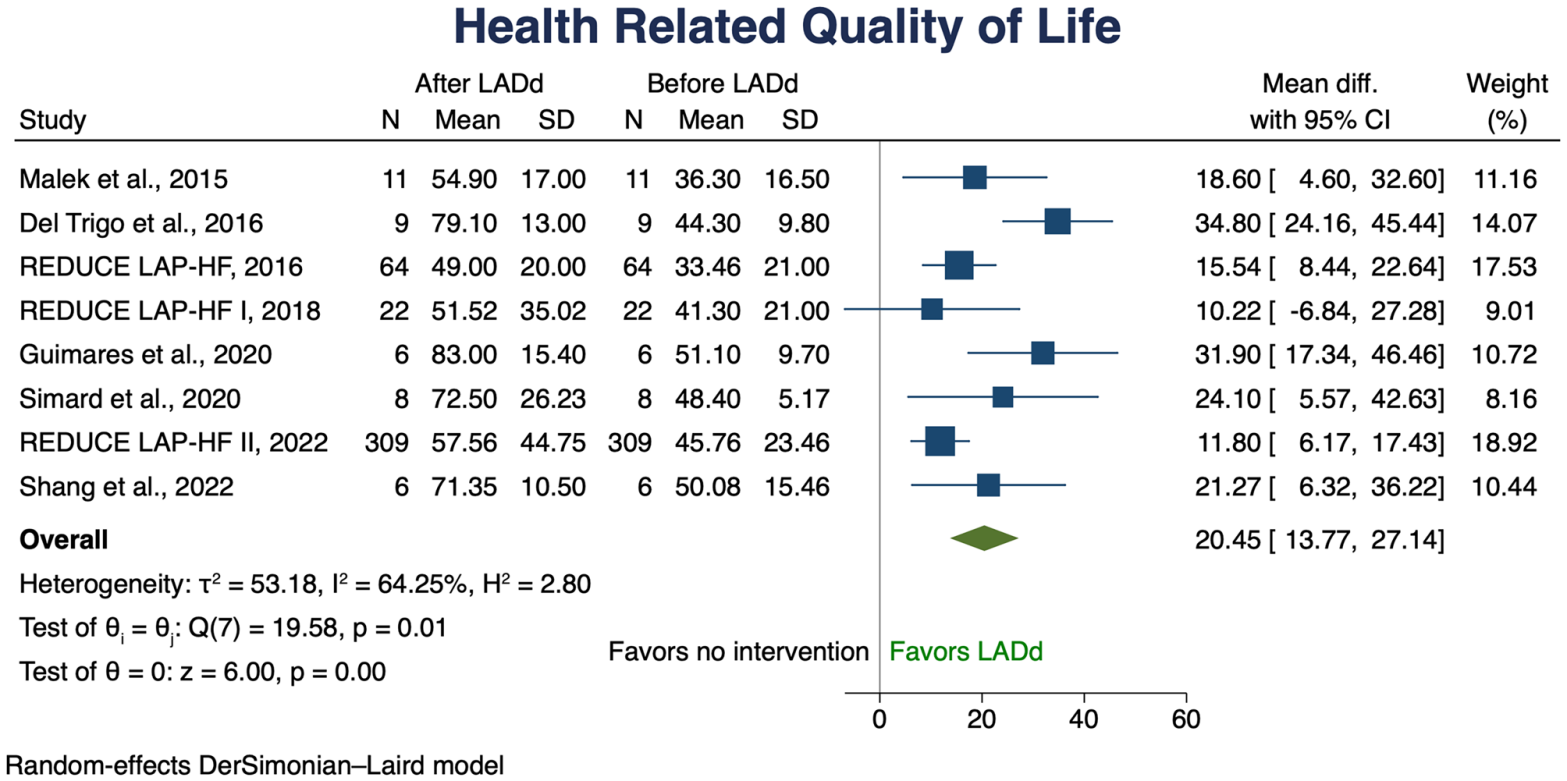
Change in HRQoL. Solid squares represent mean differences in trials and have a size proportional to the weight of the difference. The 95% confidence intervals (CI) for individual trials are denoted by lines and those for the pooled mean differences by empty diamonds CI, confidence interval; HRQoL, health-related quality of life

**Fig. 6:**
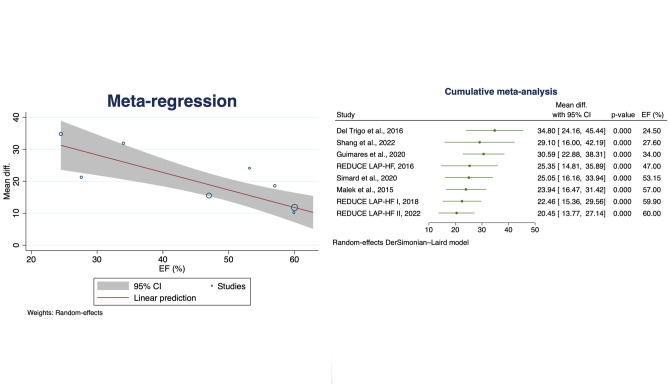
Meta regression analysis (on the left) and cumulative meta-analysis (on the right) for change in HRQoL using EF as moderator. CI, confidence interval; HRQoL, health-related quality of life

Eight studies assessed QoL, the average HRQoL score improved by 20.45 points (95% confidence interval 13.77–27.14) (Fig. 5). To further understand this finding and to assess the considerable reported heterogeneity, meta regression and cumulative meta-analysis were performed according to EF (Fig. 6). Meta regression analysis showed a consistent effect across the spectrum of EF, with a greater effect in low EFs; cumulative meta-analysis confirmed this observed trend.

There were no significant differences among the different devices in terms of 6MWD, NYHA class change, or HRQoL improvement.

### Secondary outcomes

No significant difference was found for the reduction of TAPSE, HHF, NT pro-BNP, mRAP, and mPAP (Fig. S2). This lack of statistical significance may be ascribable to the limited number of studies assessing these specific outcomes. Six studies evaluated PCWP difference; mean PCWP was reduced by 4.67 mmHg by LADd implantation (95% confidence interval: 7.59–1.75) (Fig. S3).

### Safety outcomes

**Fig. 7:**
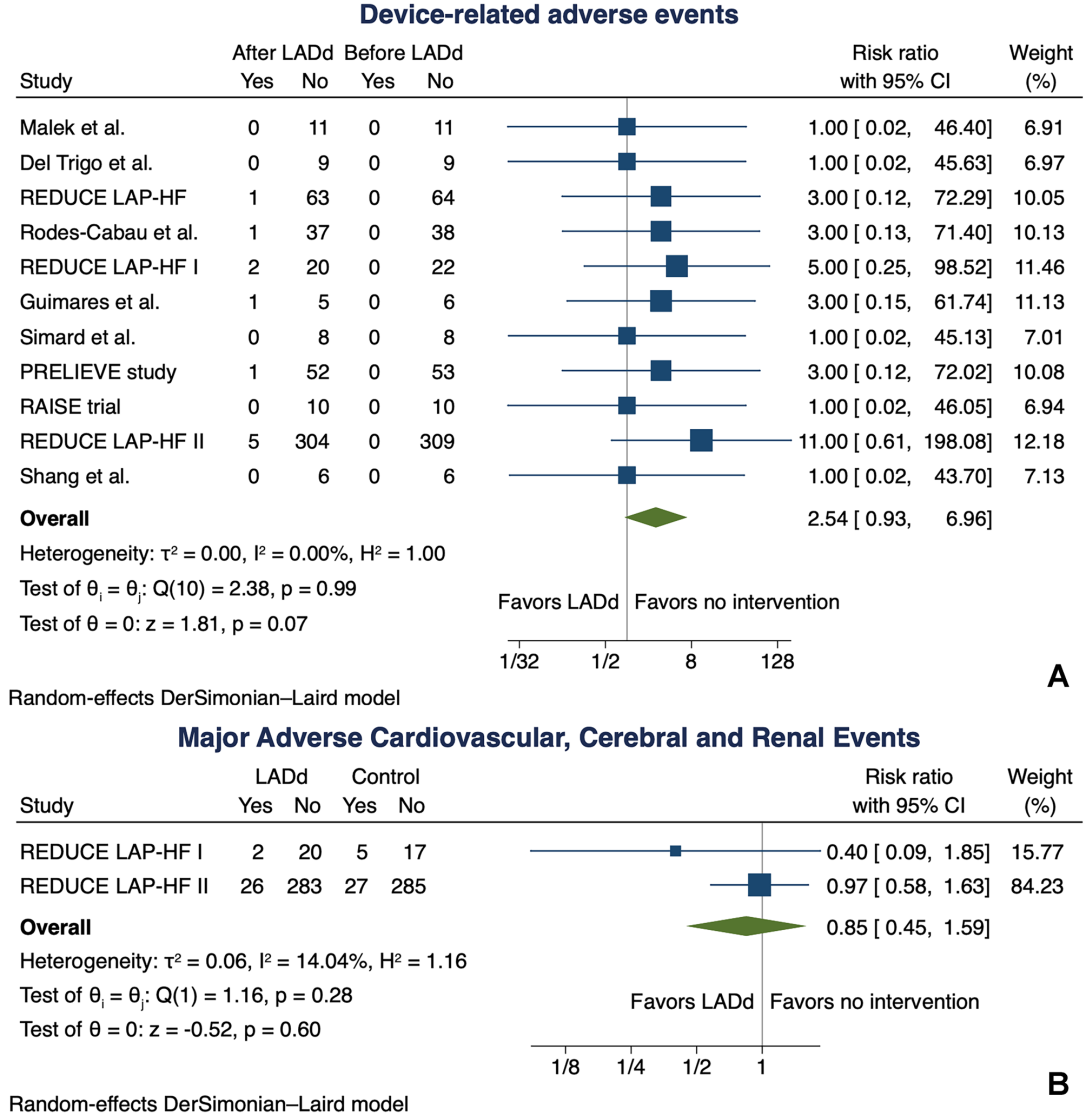
Meta-analysis for device-related adverse events **A** and Major Adverse Cardiovascular, Cerebral and Renal events **B**. CI, confidence interval

All studies examined device-related adverse events, defined as either device embolization, migration or removal, stroke (fatal and non-fatal) or transient ischemic attack, cardiac tamponade, and emergency cardiac surgery, as previously described. LADd implantation did not result in a significant higher risk of device-related adverse events (RR 2.54, 95% CI 0.93–6.96) (Fig. 7). When comparing the two RCT [17] on incidence of MACCRE, LADd implantation did not result in a significant higher risk of MACCRE (RR 0.85, 95% CI 0.45–1.59) (Fig. 7).

### Publication bias and sensitivity analysis

Publication bias for all outcomes was assessed. The Egger’s tests revealed no publication bias when corrected for meta-regression analysis moderators (Table S3). To evaluate the consistency of the findings, sensitivity analyses were conducted on all outcomes and were stable (Fig. S4).

## Discussion

The present meta-analysis of mostly non-randomized, open-label studies, by investigating the effect of different devices for left atrial decompression in 547 symptomatic patients with HF and a wide spectrum of EFs, showed that the procedure improves submaximal exercise and functional capacity assessed by 6MWD and NYHA class, and improves HRQoL. In particular, the 6MWD, a valuable index of submaximal exercise capacity, increased by approximately 15% and the NYHA class was reduced by approximately 30%. Moreover, device implantation showed a significant improvement of PCWP with no effects on other hemodynamic, prognostic, or echocardiographic endpoints (TAPSE, HHF, NT-proBNP levels, mRAP, and mPAP). There were no significant differences among devices in terms of 6MWD, NYHA class change or HRQoL improvement and device implantation was not related to significant adverse events.

LADd reduce left atrial pressure, by either creating a small artificial atrial septal defect or coronary sinus communication. The potential therapeutic benefit in HF patients of iatrogenic interatrial shunt starts from the observation that the presence of a congenital secundum atrial septal defect in patients with mitral stenosis (Lutembacher syndrome) seemed to be beneficial compared to isolated mitral stenosis, due to the ability to decompress left atrium by shunting blood to the right atrium and to the systemic veins; coronary sinus to left atrium devices similarly allow left atrium decompression, however, without the need of a septal puncture.

In all included studies, the procedural success rates were high for all LADd and most of them were patent at the end of the follow-up period (Table S4). Patients with both reduced and preserved EF were enrolled in the studies included in the present meta-analysis; in particular, the REDUCE LAP-HF II [23] concentrated on patients with preserved or mildly reduced EF, since no therapeutic approaches, apart from the recent efficacy reported for SGLT2i [2, 3], have demonstrated to improve soft and hard endpoints in this population. The pathophysiological substrate of LADd use in preserved and mildly reduced EFs starts from the concept that in HFpEF and in HF with mildly reduced EF patients, with normal or near-normal central venous pressure, but elevated left atrial pressure on efforts, left atrial decompression might be particularly beneficial to alleviate congestion and relief symptoms. However, HFpEF is a clinical entity that includes a great variety of patients with different phenotypes that often needs a personalized therapeutic approach; for this reason, many of the tested therapeutic approaches in this population failed to demonstrate a beneficial effect. Interestingly, our analysis showed that the effect of LADd on 6MWD, NYHA class and HRQoL was consistent across a wide spectrum of EFs with a greater effect in patients with lower EFs (Figs. 2, 4, and 6).

In contrast to previous meta-analysis on the same topic [10, 25], these results underline the efficacy of LAD approach in HFrEF and HFmrEF, with still some controversies in HFpEF, as already reported for most pharmacological approaches. In particular, Lauder et al. [25] reported an improvement in 6MWD, HRQoL, and PCWP, with better outcomes for 6MWD in patients with EF > 40%. In a subsequent meta-analysis, Yi and colleagues [10] reported that the improvement in PCWP was consistent in HF patients, with no difference regarding EF. The reason why a third meta-analysis on the topic was needed after the publication of the REDUCE LAP-HF II trial [23] is related to the clear interest in this specific subject among HF specialists and interventional cardiologists, and, especially, to some new elements that are present in our study. In particular, the inclusion of more recent trials, doubling the total number of patients compared to the aforementioned meta-analyses, and the use of EF as continuous and not dichotomic moderator in meta-regression analyses significantly impacted on meta-analyses results and study data interpretation; thus, adding these components, we demonstrated that the patient-centric outcomes improve across a wide range of EF, with better results in patients with lower EF.

Regarding quality of life, HRQoL score increased by 20.45 points; in general, a shift of 5 points is considered a small, but clinically significant change, while a change of more than 20 points is interpreted as a large significant change [26], as observed in our analysis. The HRQoL is drastically impaired in HF patients; in the studies included in the present meta-analysis, HRQoL was measured using either the MLHFQ or KCCQ, that are both robust and reliable disease specific HRQoL measurements in HF [27, 28]. The gain in HRQoL obtained after LADd implantation overcome those of most approved pharmacological HF treatments. In the DAPA-HF trial, dapagliflozin improved the KCCQ total symptom score by 7.0 points in HFrEF diabetic patients and by 5.4 points in non-diabetics at 8 months [29], whereas, in the recently published DELIVER trial [3], dapagliflozin improved the KCCQ total symptom score by 2.4 points at 8 months in HFpEF subjects. In the EMPEROR-Preserved [2] and EMPEROR-Reduced [30] trials, empagliflozin improved KCCQ by 4.5 points and 5.8 points at 12 months, respectively in HFpEF and HFrEF patients. Similar benefits on HRQoL were only recorded for transvenous edge-to-edge mitral valve repair of secondary mitral regurgitation, in which the KCQQ score increased by 12.50 points [31].

As regards safety concerns, despite different analyzed outcomes among the included studies, no differences in the occurrence of device-related adverse events were found.

Specifically, analyzing the reported adverse outcome in the REDUCE LAP-HF trial [16], at 12-month follow-up, one patient died for combined pneumonia and renal failure and one patient had a fatal stroke. In the REDUCE LAP-HF I trial [17], across the 12 months of follow-up, no device embolization, occlusion, or migration was observed; a second procedure for device removal or occlusion was never required, and no strokes, transient ischemic attacks or atrial fibrillation/atrial flutter events were reported in the device treated arm. The cumulative incidence of major adverse cardiac, cerebrovascular, and renal events was comparable in the device and in the control group (Log-rank *p* = 0.20). In the REDUCE LAP-HF II trial [17] the composite safety endpoint of cardiovascular mortality, non-fatal stroke, worsening kidney function, major cardiac events, thrombo-embolic complications, new persistent or permanent atrial fibrillation or atrial flutter, and ≥ 30% increase in right ventricular size or ≥ 30% decrease in TAPSE were not significantly different between the device and sham-controlled arms (*p* = 0.11). Rodes-Cabau et al. [18] reported only one patient experiencing a cardiac tamponade during the in-hospital stay, whereas Guimares et al. [19] reported one patient with advanced HF dying due to an electrical storm. In the PRELIEVE study [21] only one patient experienced post-procedural bleeding and syncope. In the studies by Del Trigo et al. [15], Simard et al. [20], Shang et al. [24], Malek et al. [14], and in the RAISE trial [22], no device-related adverse events were recorded at follow-up. In the present meta-analysis, we analyzed both a composite of averse-related outcomes and a composite of major adverse cardiovascular, cerebral, and renal events just considering the data from RTCs; for both safety outcomes, LADd implantation was neutral.

Thus, LADd implantation is a safe procedure and provide a functional benefit in HF patients, with better evidence in patients with lower EFs. However, despite the benefits of LADd therapy, the present findings should be interpreted with caution until verified by larger, appropriately powered, randomized trials examining the impact on hard endpoints in HFrEF.

## Limitations

Our meta-analysis suffers from some limitations. First, most of the included studies were small, single-arm feasibility studies without control groups and with variable follow-up durations. Thus, we cannot rule out non-specific therapeutic effects, such as the placebo effect. Moreover, concerns may exist about the generalizability of our findings due to the limited sample size and considerable heterogeneity of some outcomes. Meta regression analysis according to EF proved to be a significant explanator for the reported heterogeneity. Although the improvements in clinical outcomes, such as 6MWD, NYHA class, and HRQoL are encouraging, they need to be validated by appropriately powered and, ideally, sham-controlled randomized trials. Several trials are now active, like the ALt FLOW US (NCT03523416) [32], RELIEVE-HF (NCT03499236) [33], and PROLONGER [34], recruiting patients with both HFrEF and HFpEF. Second, this meta-analysis is not based on individual patient data. Third, functional outcomes, including the 6MWD, are not simply reliant on cardiopulmonary conditions.

## Conclusions

LADd implantation in HF patients is a feasible and safe procedure, associated with considerable improvements in patient-centric outcomes, such as submaximal exercise capacity, NYHA class, and HRQoL across a wide spectrum of EF, with better outcomes in patients with more impaired systolic function. These findings need confirmation by larger sham-controlled trials.

## Supplementary Information

Below is the link to the electronic supplementary material.

Supplementary file1 (DOCX 20896 KB)

## Data Availability

All data and STATA.do files will be available at reasonable request to the corresponding author.
