# Supplementary material for: Left atrium decompression devices across the spectrum of ejection fraction in heart failure: an updated systematic review and meta-regression analysis
Source: Heart Fail Rev. 2023 May 10;28(5):1151–61. doi: 10.1007/s10741-023-10317-2 (PMC10403397; doi:10.1007/s10741-023-10317-2)

Title:

**Left atrium decompression devices across the spectrum of ejection fraction in heart failure: an updated systematic review and meta-regression analysis**

Authors:

Christian Basile^a^ MD, Stefania Paolillo^a^ MD PhD, Paola Gargiulo^a^ MD PhD, Federica Marzano^b^ MS PhD, Santo Dellegrottaglie^c^ MD PhD, Vincenza Abbate^a^ MD, Antonio Ambrosio^a^ MD, Francesca Carbone^a^ MD, Simona Dell’Aversana^a^ MD, Immacolata Esposito^a^ MD, Maria Francesca Fierro^a^ MD, Pasquale Perrone-Filardi^a,d^ MD PhD

Affiliations:

^a^ Department of Advanced Biomedical Sciences, Federico II University of Naples, Naples, Italy

^b^ IRCCS Synlab SDN S.p.a., Naples, Italy

^c^ Villa dei Fiori Clinic, Corso Italia, I-80011, Acerra, Naples, Italy

^d^ Mediterranea Cardiocentro, Naples, Italy

**Page 2:** Research methods

**Page 3, 4 and 5:** Table S1

**Page 6:** Table S2

**Page 7:** Table S3

**Page 8:** Table S4

**Page 9:** Figure S1

**Page 10:** Figure S2

**Page 11:** Figure S3

**Page 12:** Figure S4

**Research methods:**

PUBMED:

(Interatrial shunt devices) OR (atrial flow regulators)

WEB OF SCIENCE:

Interatrial shunt devices (all Fields) OR atrial flow regulators (All Fields)

**Table S1:** PRISMA checklist

| **TITLE** | | | **Reported on page** |
| --- | --- | --- | --- |
| Title | 1 | Identify the report as a systematic review, meta-analysis, or both. | 1 |
| **ABSTRACT** | | | |
| Structured summary | 2 | Provide a structured summary including, as applicable: background; objectives; data sources; study eligibility criteria, participants, and interventions; study appraisal and synthesis methods; results; limitations; conclusions and implications of key findings; systematic review registration number. | 3 |
| **INTRODUCTION** | | | |
| Rationale | 3 | Describe the rationale for the review in the context of what is already known. | Performed, page 4 |
| Objectives | 4 | Provide an explicit statement of questions being addressed with reference to participants, interventions, comparisons, outcomes, and study design (PICOS). | Performed, page 4 |
| **METHODS** | | | |
| Protocol and registration | 5 | Indicate if a review protocol exists, if and where it can be accessed (e.g., Web address), and, if available, provide registration information including registration number. | Performed, page 4 |
| Eligibility criteria | 6 | Specify study characteristics (e.g., PICOS, length of follow-up) and report characteristics (e.g., years considered, language, publication status) used as criteria for eligibility, giving rationale. | Performed, page 4, 5 |
| Information sources | 7 | Describe all information sources (e.g., databases with dates of coverage, contact with study authors to identify additional studies) in the search and date last searched. | Performed, page 4, 5, 6 |
| Search | 8 | Present full electronic search strategy for at least one database, including any limits used, such that it could be repeated. | Performed, page 4, 5, 6 and Appendix |
| Study selection | 9 | State the process for selecting studies (i.e., screening, eligibility, included in systematic review, and, if applicable, included in the meta-analysis). | Performed, page 5, 6, Figure 1 |
| Data collection process | 10 | Describe method of data extraction from reports (e.g., piloted forms, independently, in duplicate) and any processes for obtaining and confirming data from investigators. | Performed, page 5, 6 |
| Data items | 11 | List and define all variables for which data were sought (e.g., PICOS, funding sources) and any assumptions and simplifications made. | Performed, page 5, Table 1 |
| Risk of bias in individual studies | 12 | Describe methods used for assessing risk of bias of individual studies (including specification of whether this was done at the study or outcome level), and how this information is to be used in any data synthesis. | Performed, page 5, 6, Appendix |
| Summary measures | 13 | State the principal summary measures (e.g., risk ratio, difference in means). | Performed, page 5, 6 |
| Synthesis of results | 14 | Describe the methods of handling data and combining results of studies, if done, including measures of consistency (e.g., I^2^) for each meta-analysis. | Performed, page 5, 6 |
| Risk of bias across studies | 15 | Specify any assessment of risk of bias that may affect the cumulative evidence (e.g., publication bias, selective reporting within studies). | Performed, page 5 |
| Additional analyses | 16 | Describe methods of additional analyses (e.g., sensitivity or subgroup analyses, meta-regression), if done, indicating which were pre-specified. | Performed, page 6 |
| **RESULTS** | | | |
| Study selection | 17 | Give numbers of studies screened, assessed for eligibility, and included in the review, with reasons for exclusions at each stage, ideally with a flow diagram. | Performed, page 6 and Figure 1 |
| Study characteristics | 18 | For each study, present characteristics for which data were extracted (e.g., study size, PICOS, follow-up period) and provide the citations. | Performed, Table 1 |
| Risk of bias within studies | 19 | Present data on risk of bias of each study and, if available, any outcome-level assessment (see Item 12). | Performed, Appendix |
| Results of individual studies | 20 | For all outcomes considered (benefits or harms), present, for each study: (a) simple summary data for each intervention group and (b) effect estimates and confidence intervals, ideally with a forest plot. | Performed, figure 2 and 3 |
| Synthesis of results | 21 | Present results of each meta-analysis done, including confidence intervals and measures of consistency. | Performed, page 6, 7 |
| Risk of bias across studies | 22 | Present results of any assessment of risk of bias across studies (see Item 15). | Performed, appendix |
| Additional analysis | 23 | Give results of additional analyses, if done (e.g., sensitivity or subgroup analyses, meta-regression [see Item 16]). | Performed, page 6, 7 |
| **DISCUSSION** | | | |
| Limitations | 25 | Discuss limitations at study and outcome level (e.g., risk of bias), and at review level (e.g., incomplete retrieval of identified research, reporting bias). | Full discussion page 7, 8, 9 |
| Conclusions | 26 | Provide a general interpretation of the results in the context of other evidence, and implications for future research. | Full discussion, page 9 |
| **FUNDING** | | | |
| Funding | 27 | Describe sources of funding for the systematic review and other support (e.g., supply of data); role of funders for the systematic review. | Performed |

**Table S2:** Quality of evidence according to the GRADE approach.

|  | **Quality Assessment** | | | | | | |
| --- | --- | --- | --- | --- | --- | --- | --- |
| **Outcomes** | **N Studies (Participants)** | **Risk of bias** | **Inconsistency** | **Indirectness** | **Imprecision** | **Publication Bias** | **Quality of the evidence (GRADE)** |
| **6MWD** | 10 (227) | Low risk | Low risk | Low risk | Low risk | Undetected | ++++ (high) |
| **NYHA** | 11 (547) | Low risk | Low risk | Low risk | Low risk | Undetected | ++++ (high) |
| **HRQoL** | 8 (435) | Low risk | Low risk | Low risk | Moderate risk | Undetected | +++ (moderate) |
| **HHF** | 5 (414) | Moderate risk | Low risk | Low risk | Low risk | Undetected | +++ (moderate) |
| **NT-proBNP** | 7 (133) | Low risk | Low risk | Low risk | Low risk | Undetected | ++++ (high) |
| **mRAP** | 6 (136) | Low risk | Low risk | Low risk | Low risk | Undetected | ++++ (high) |
| **PCWP** | 6 (136) | Low risk | Low risk | Low risk | Low risk | Undetected | ++++ (high) |
| **mPAP** | 4 (119) | Low risk | Moderate risk | Low risk | Low risk | Undetected | +++ (moderate) |
| **TAPSE** | 4 (117) | Low risk | Low risk | Low risk | Moderate risk | Undetected | +++ (moderate) |

**Table S3:** Egger test p-value for the evaluated outcomes.

| Outcome | Egger test p-value | Egger test p-value corrected for EF |
| --- | --- | --- |
| 6MWD | 0.7568 | 0.3184 |
| NYHA class improvement | **0.0011** | 0.1274 |
| HRQoL | 0.3629 | 0.4226 |
| mPCWP | **0.0001** | 0.1263 |

**Table S4:** Procedural success and shunt patency.

| Author | Year | Successful LADd implantation, n (%) | No. of patients with patent shunts / No. of patients undergoing assessment for shunt patency  at 12 months |
| --- | --- | --- | --- |
| Malek et al. | 2015 | 11 (100%) | 11 (100%) |
| Del Trigo et al. | 2016 | 9 (100%) | NA |
| REDUCE LAP-HF | 2018 | 64 (97%) | 54/54 (100%) |
| Rodes-Cabau et al. | 2018 | 38 (100%) | 18/36 (50%) |
| REDUCE LAP-HF I | 2018 | 20 (91%) | 20/20 (100%) |
| Guimares et al. | 2020 | 10 (100%) | 6/6 (100%) |
| Simard et al. | 2020 | 8 (73%) | NA |
| PRELIEVE study | 2021 | 53 (95%) | 45/49 (92%) |
| RAISE trial | 2022 | 10 (100%) | NA |
| REDUCE LAP-HF II | 2022 | 309 (98%) | NA |
| Shang et al. | 2022 | 6 (100%) | NA |

**Figure S1:** PRISMA 2020 flow diagram for new systematic reviews.

**
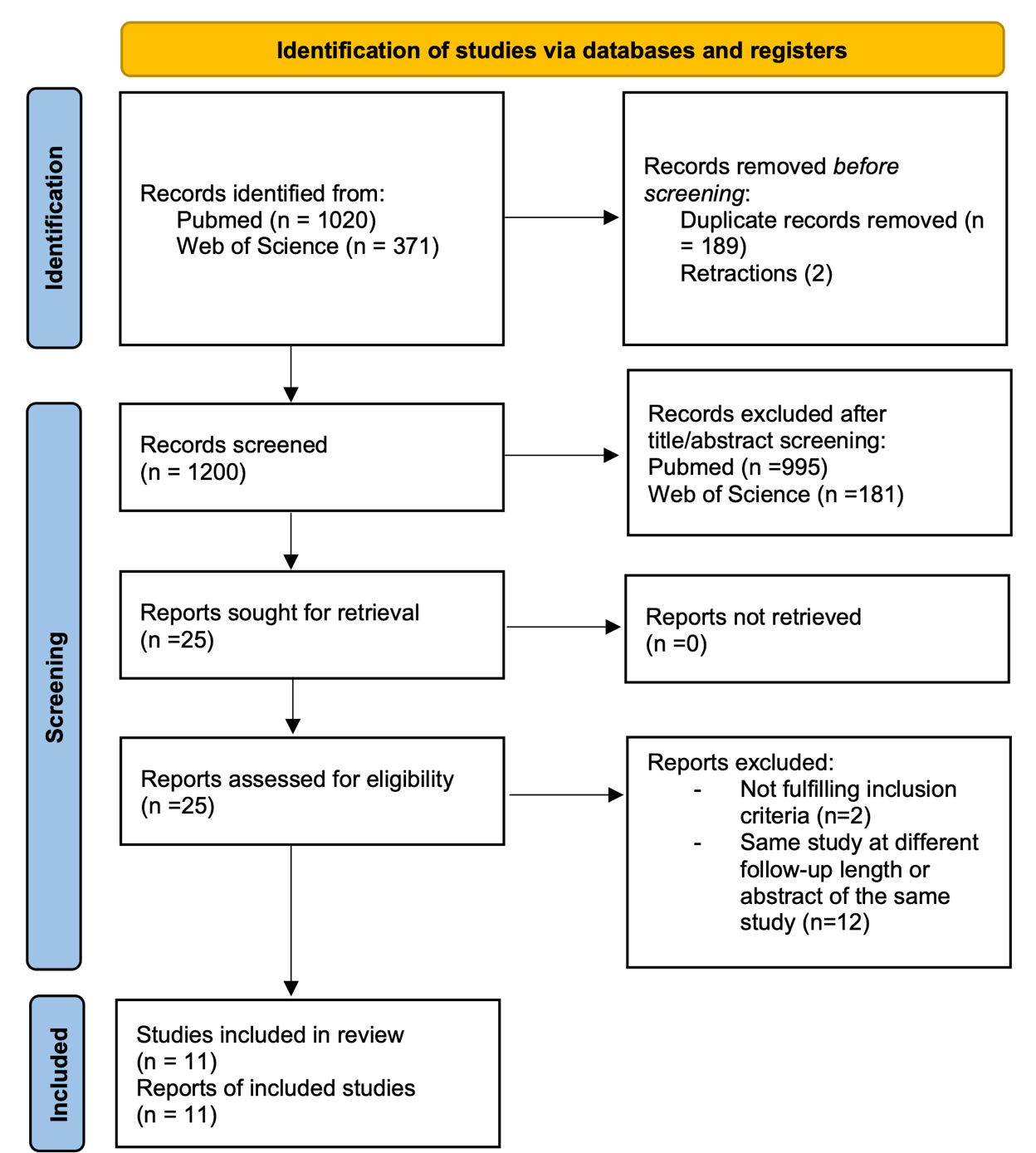
**

**Figure S2:** Meta-analysis for change in TAPSE (A), HHF (B), Changes in NTproBNP (C), PAPs (D) and RAP (E). CI, confidence interval.

**
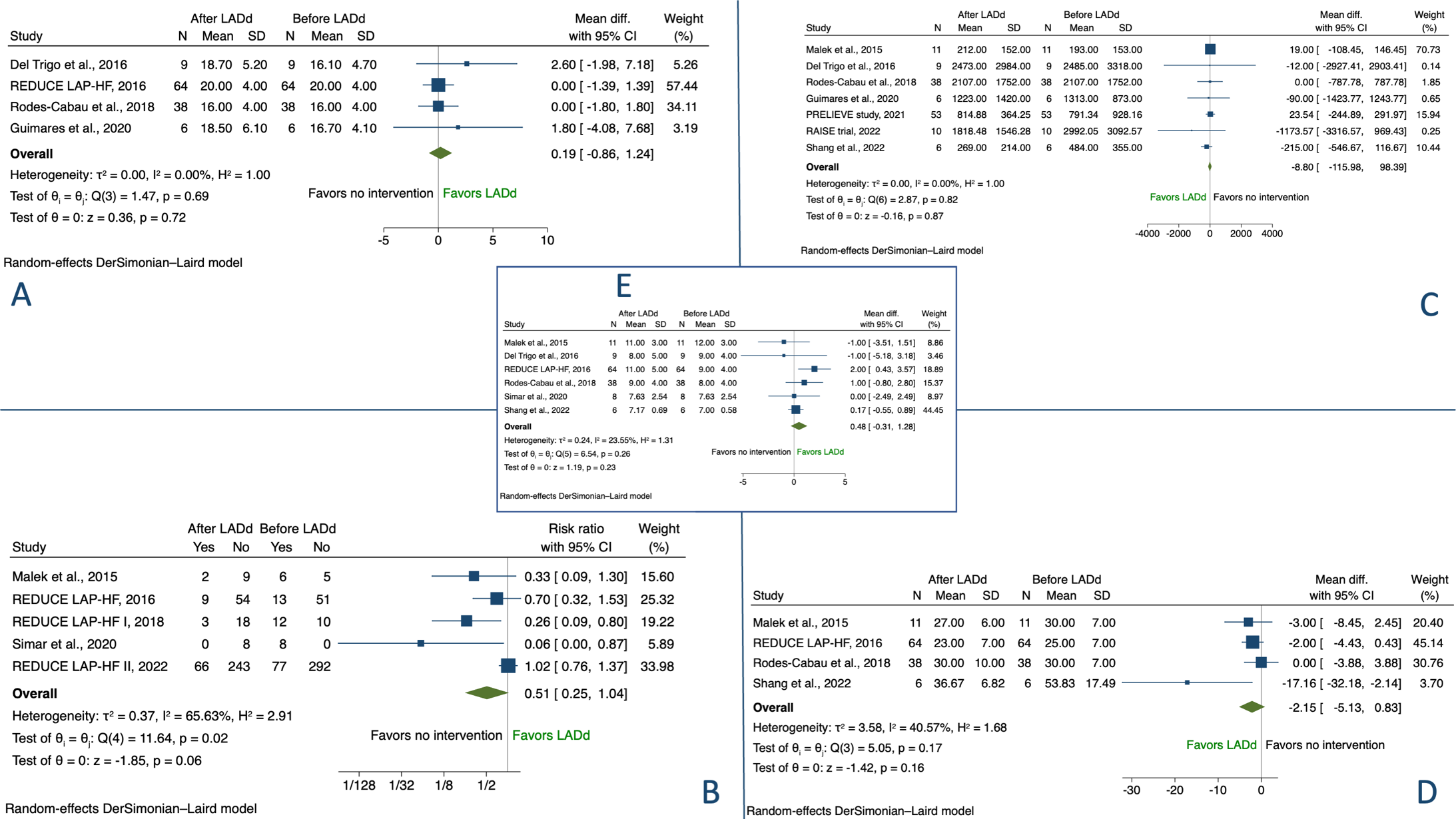
**

**Figure S3:** Change in pulmonary capillary wedge pressure expressed in mmHg. Solid squares represent mean differences in trials and have a size proportional to the weight of the difference. The 95% confidence intervals (CI) for individual trials are denoted by lines and those for the pooled mean differences by empty diamonds. CI, confidence interval; PCWP, pulmonary capillary wedge pressure.

**Figure S4:** Sensitivity analysis through the leave-one-out meta-analysis method for change in NYHA class (A), HRQoL (B), PCWP (C) and 6MWD (D). CI, confidence interval.


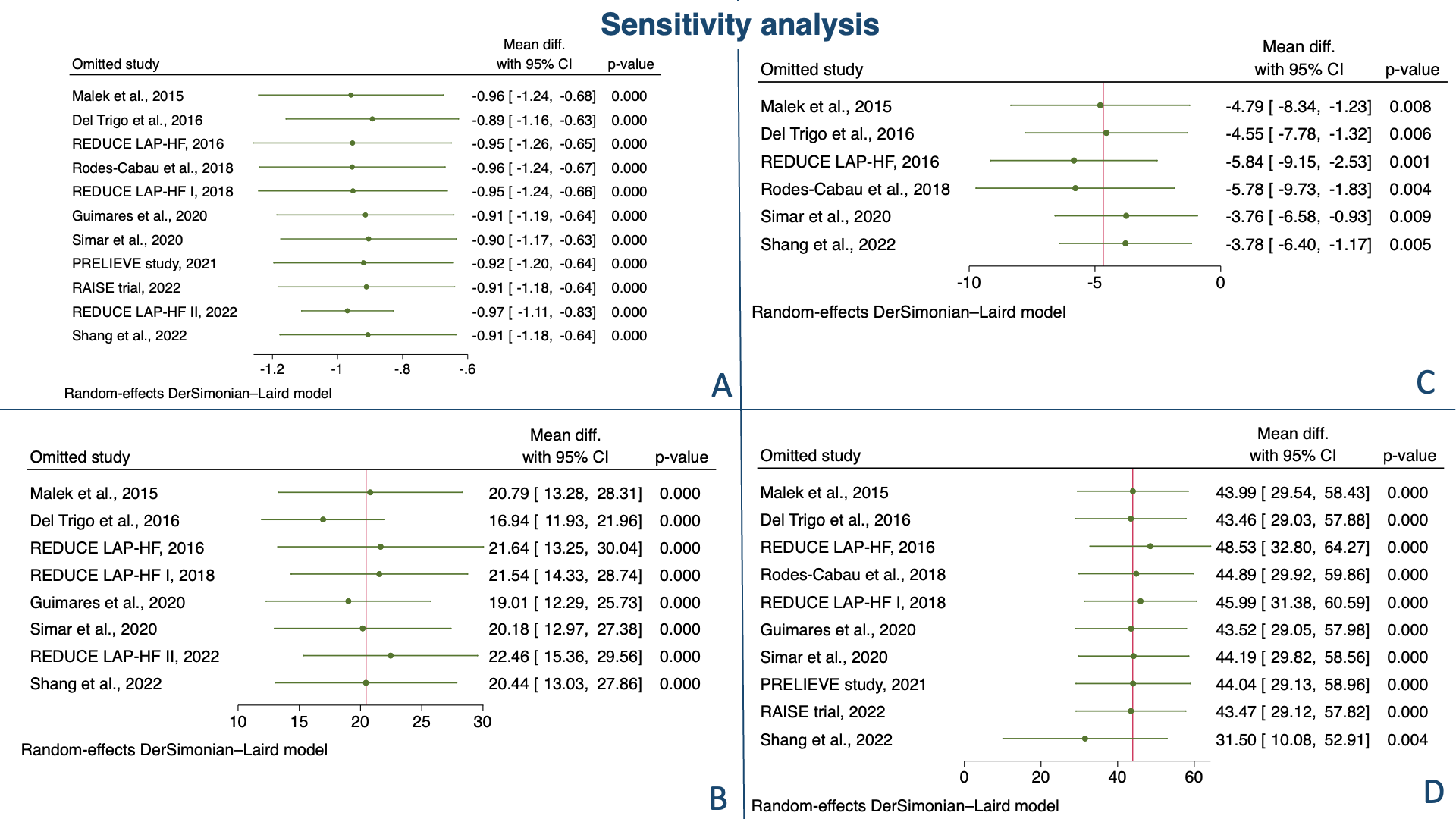

Supplement: Supplementary file 1 — Supplementary file1 (DOCX 20896 KB) [file 10741_2023_10317_MOESM1_ESM.docx]
